# Inclusive education pandemic: Learning barriers for children with disabilities in South Africa

**DOI:** 10.4102/ajod.v13i0.1462

**Published:** 2024-12-06

**Authors:** Raymond Chirowamhangu

**Affiliations:** 1 Department of Sociology, Faculty of Humanities, University of Pretoria, Pretoria, South Africa

**Keywords:** Right to a basic education, inclusive education, children with disability, policy, South Africa

## Abstract

**Background:**

Children with disabilities encounter obstacles attaining basic education. Significantly, previous studies on South Africa have shown that up to 70% of the children with disabilities are out of school. Despite efforts to support inclusive education through White Paper 6 policy, the deployment of resources and transformation of the education sector has been a slower process.

**Objectives:**

The main objective of the article is to explore the challenges of basic education faced by children with disabilities in South Africa.

**Method:**

The study was conducted during the COVID-19 pandemic using a qualitative research methodology. The data were collected using key informant interviews through online media platforms. The data analysis was conducted using computer-aided software in the form of ATLAS.ti 8.

**Results:**

This study established several challenges faced by special needs schools, especially in the rural areas. These include a limited number of special needs schools, scholar transport, enrolment, lack of psychosocial and expert support, sanitation and infrastructure and the impact of COVID-19 pandemic on children with disabilities.

**Conclusion:**

The article concludes that even though White Paper 6 focusses on Special Needs Education in South Africa, there remains poor policy implementation to ensure inclusivity for learners with disabilities.

**Contribution:**

The research provides an understanding of the challenges faced by children with disabilities to assist policy makers with recommendations and areas of concern to improve policy implementation of the White Paper 6 in South Africa.

## Introduction

The right to basic education is universal, and its realisation is mandatory for all children (Strohwald 2021). The basis of children’s rights, specifically the rights of children with disabilities, is entrenched in the Convention on the Rights of Persons with Disabilities (CRPD), African Charter on the Rights and Welfare of the Child (ACRWC) and Convention on the Rights of the Child (CRC). In addition, basic education is recognised as a global development priority through the Education for All (EFA) initiative and the United Nations Sustainable Development Goal (SDG) 4 to promote inclusive and equitable quality education for all children.

The rights of children with disabilities are violated when they are excluded from the learning process and adequate services to support their education (Musenyente, Han & Knigge 2022). Similarly, the African Committee of Experts on the Rights and Welfare of the Child (ACERWC) reveals that especially in the rural areas of Africa, there are disparities in school enrolment and attendance of children with special needs (ACERWC 2022). Special needs schools provide appropriate support for low-, medium- and high-level needs of learners to offer maximum support for learners experiencing challenges (Directorate Inclusive Education 2014; eds. Khumalo & Hodgson 2017; Mahlaule, McCrindle & Napoles 2024). The focus on special needs schools in this research is based on the lack of progress in the implementation of inclusive education in the post-democracy era of South Africa (Engelbrecht 2006). Using the case study of South Africa, the basic education challenges on children with disabilities are exposed in this research.

### Basic education perspectives: Constitution versus Reality

The classical writings of the *Pure Theory of Law* by Hans Kelsen underline that the constitution is the link between ‘government and the governed’ by instituting laws and policies (Owosuyi 2016). However, as presented in this research, the application of rights outlined in the constitution is not all inclusive, especially for children with disabilities. As supported in an opinion critique by Liebenberg (2017), the constitution is a model for human rights but in reality, these are only enjoyed by a selective few.

The rights of children are outlined in Section 28 of the Constitution, which reinforces that the child’s bests interests are paramount in all matters concerning the child (Republic of South Africa 1996). Fundamentally, in relation to this research, the right to basic education is pronounced in Section 29(1) of the Constitution, but as contested by other researchers, there is no legal clarity on the quality of education (Donohue & Bornman 2014). Thus, to overcome that deficiency and to establish the parameters of this study, the researcher adopts the perspective of McConnachie, Skelton and McConnachie (2016), which states that basic education represents two important aspects. Firstly, ‘basic’ is interpreted as the education level computed based on time; secondly, ‘education’ focusses on the learning content, for instance, arithmetic reading and skills (McConnachie et al. 2016). This definition is in sync with the World Declaration on Education for All, which focusses on minimum levels of literacy, numeracy and essential life skills (United Nations, Educational, Scientific and Cultural Organization [UNESCO] 1990).

This furthermore prompts the need for detailed policy frameworks, such as the *South African Schools Act 84 of 1996*, which is the first port of call on contextualising basic education by making it compulsory for all children from the age of 7 to 15 years or the equivalent Grade 9, whichever comes first (Republic of South Africa 1996; Strohwald 2021). This legal instrument is an architect of making basic education available, accessible and affordable for every child. The Act also contains regulations relating to the minimum uniform norms and standards for public school infrastructure, which outline acceptable and adequate measures for access to basic education. The regulations attempt to provide a timeline to eradicate school infrastructure backlogs and legal binding standards for provincial education governments to be held accountable (Gamede 2020).

Consequently, the Juma Musjid legal case in South Africa underpins the nature and context of basic education (Juma Musjid v Essay 2011). The case that was between Juma Musjid primary school and the Department of Education is significant because it was the first time the Constitutional Court of South Africa considered the right to basic education in relation to the best interests of the child principle. This results from the failure of the Constitution on providing an exact definition of basic education (Arendse 2020). The case judgement aligned with Article 29 of the CRC and Section 29 of the Constitution, which focussed on the right to basic education. The Musjid case concluded that ‘access to a school’ is a necessity for achieving the right to basic education (Juma Musjid v Essay 2011).

### Contextualisation of inclusive education

Shikwambana and Fourie (2023) highlight that inclusive education is concerned with removing all barriers to learning and promoting the participation of all students, including those vulnerable to exclusion and marginalisation. Furthermore, Murungi (2015) states that the term is mainly applied in the context of persons with disabilities to promote access to education in society.

The Universal Declaration of Human Rights adopted in 1948 declares that ‘everyone has the right to education’ (United Nations 1948:7). The provisions of Articles 2(2), 23(1) of the CRC and Article 13(1) of the ACRWC demonstrate that all children with special needs have the right to education without discrimination on any grounds (ACRWC 1990; CRC 1989). The focus on inclusive education is elaborated further in Article 24(2)(a) of the CRPD, which calls for state parties to ensure a non-discriminatory, inclusive education system in which:

Persons with disabilities are not excluded from the general education system on the basis of disability, and that children with disabilities are not excluded from free and compulsory primary education, or from secondary education, on the basis of disability. (CRPD 2006:17)

Although most countries seem to share the same ideology and commitment towards education for learners with special needs, the implementation of inclusive education has not been successful (Mokaleng & Möwe 2020). For example, the CRPD gives specific guidelines to promote inclusive education, such as, using braille, alternative script, augmentative and alternative modes of teaching, but this has not been the reality, especially in rural schools in South Africa. Moreover, the high level of exclusion is a culmination of several factors, including, limitations in policy, no strategies of delivery and the erroneous assumption that inclusive education is expensive to implement (Gow, Mostert & Dreyer 2020).

Thus, Ssenyonjo (2017) reiterates that the non-domestication of regional and international treaties perpetuates the violation of rights on children with disabilities. In response, the Government of South Africa embedded the CRPD into the White Paper 6 which specifically addresses Special Needs Education within a policy framework (Walton & Engelbrecht 2022). The White Paper 6 is the key framework for supporting an inclusive education system in South Africa. It is worth reinforcing that White Paper 6 is not a law but rather a policy document that helps the Government to account for specific deliverables on inclusive education. The policy provides a broader scope to address the diverse needs of learners who experience learning barriers.

The historical context of South Africa is significant because the education system during apartheid was discriminative, not only based on race but also for children with disabilities (Deghaye 2021). Scholarly research in South Africa has explored the lack of inclusive education for learners with disabilities, especially in the rural areas, because of the scarcity of resources (Banks et al. 2022; Chirowamhangu 2023). Therefore, inclusive education seeks to confront exclusionary policies that violate children’s rights and result in excessive discrimination. Achieving inclusive and equitable quality education for all requires increasing efforts, especially in sub-Saharan Africa for vulnerable populations, including persons with disabilities and poor children in rural areas. State parties are expected to remove legislative and policy barriers to the inclusion of children in primary and secondary education and repeal any existing legislation that define any group of children with disabilities as ‘ineducable’.

#### Matland’s top-down implementation approach to White Paper 6 policy

South Africa’s inclusive education policy has been known as ‘symbolic implementation’ because of failure and non-implementation, such as the case of the White Paper 6 that was viewed an important intervention when published in 2001, but the implementation has yielded little success in the Eastern Cape province (Adewumi, Mosito & Agosto 2019). In addition, Walton and Engelbrecht (2022) highlight that the poor implementation of inclusive education policy in South Africa is because of the lack of political will. Specifically, the Eastern Cape Department of Education has been characterised by politicisation of the allocation of resources (Kota et al. 2018). For example, a study on the Eastern Cape found that it was the fastest South African province to reach a million coronavirus disease 2019 (COVID-19) vaccinations, but when it comes to issues related to education, the province is still plagued by the poor education infrastructure and the highest number of pit latrine toilets (Chirowamhangu 2023).

Literature highlights the lack of clarity on the extent of inclusiveness in the White Paper 6 policy document (Hudson, Hunter & Peckham 2019). In support of this view, Makoelle and Burmistrova (2020) reveal that there remain many school-level and cultural barriers to inclusion. For instance, scholars show that with regard to cultural barriers children with disabilities are discriminated because disability is attributed to family sin, witchcraft and conflict with the ancestors (Ndlovu 2023; Yaacob et al. 2021). This makes it difficult to implement a bottom-up approach because of the diversity of cultural, social and religious views on disability. Another illustration is based on the economics of implementing the policy, as Dehnhardt, Grothmann and Wagner (2022) purport that the policy yields cost-effective benefits. But as challenged in recent studies, this seems unrealistic in South Africa considering the substantial increase in provincial funding (Walton & Engelbrecht 2022). Furthermore, the Republic of South Africa (2022) shows that the Department of Education in the Eastern Cape province failed to implement initiatives aligned with White Paper 6 because it was underfunded. Thus, the top-down approach helps to streamline the objectives of the policy, actions needed, delegation of duties to ensure children with disability have access to basic education.

Therefore, Matland’s top-down approach is instrumental in policy implementation because it provides clarity on the goals of the policy (Hudson et al. 2019). This means the government and policy makers articulate fundamental principles, guided by specific mandates, and establish a mechanism to account for achieving the policy. This calls for clear and effective legislative frameworks to support inclusive education in South Africa (Ramango & Naicker 2022). In the process, this helps to eradicate negative attitudinal perspectives from legislators, educators and learners to foster inclusive participation of children with disabilities in the mainstream school system. In a similar case study on inclusive education in the Eastern Cape province, the researchers concluded that there was lack of policy coherence and accountability between the provincial Department of Education and the National Department of Education (Bipath, Tebekana & Venketsamy 2021). However, Shukia (2020) argues that the approach fails to recognise the complex implementation structures, thus, giving policy makers exclusive control even when they lack proper understanding of the realities faced by persons with disabilities.

## Research methods and design

### Study design

The study was conducted from May to July 2020, at the peak of COVID-19 pandemic. In response, the Government of South Africa, through the *Disaster Management Act*, introduced lockdown restrictions to contain and mitigate the virus (Republic of South Africa 2020). The study adopted a qualitative research design anchored on an interpretive paradigm. Scholars support this approach as it allows diverse findings rather than having imposed dominant interpretations (Kumar 2019; Thanh & Thanh 2015). A qualitative research approach seeks an in-depth, holistic understanding of people’s lived experiences within their natural environment (Flick 2018). The qualitative design is flexible, responsive and provides detailed descriptions of the issues being investigated (Creswell & Creswell 2022; Shikwambana & Fourie 2023). This methodology contextualises the realities of education challenges experienced by children with disabilities in the Eastern Cape province.

### Study setting

The Eastern Cape province of South Africa has the second highest rate of child poverty at 79%, with an education system described as an ‘education crisis’ engraved in poverty, failed social welfare reforms and poor infrastructure (Ngumbela 2021; Stats SA 2020:7). In addition, more than 60% of its populace reside in rural areas (Stats SA 2019). This not only shows the extent of poverty in the area but also anchors the research on a demography, which is often overlooked, the rural population. The study was conducted in the Nelson Mandela Bay and Buffalo City municipalities, covering the Amathole, Sarah Baartman, Chris Hani and OR Tambo districts. This included areas such as, Gqeberha, East London, Qonce, Dikeni, Middeldrift, Mthatha, Port Alfred, Komani, Seymour and Fort Beaufort.

### Study population and sampling

The research population comprised 50 representatives. The breakdown of participants included 20 special needs schools, 15 non-governmental organisations (NGOs), 10 community leaders and 5 Government departments from the Eastern Cape province. The participants were selected using the non-probability method in the form of snowball sampling. Kirchherr and Charles (2018) describe snowball sampling as the process by which one interviewee gives the researcher the name of at least one more potential interviewee. Thus, using this method, the probability of selecting the participants was known by the researcher (Kumar 2019). It also helped to ensure that the participants were competent with the relevant knowledge on the issues discussed in the research. The method was applied in all the categories of participants. For example, the interviews representatives from the Department of Basic Education would provide contacts of participants working on other programmes, such as psychosocial support that works with learners with special needs. Similarly, because many of the NGOs have a network that they use when lobbying for the rights of children with disabilities, it was easier to get recommendations on NGOs, which could assist with the interviews.

### Data collection

Data were collected using semi-structured, key informant interviews. This supported the integration of open-ended questions to allow for an honest and open exchange, whereby the participants responded based on their own perspectives and experiences (Busetto, Wick & Gumbinger 2020). Because of the COVID-19 restrictions, some interviews were conducted telephonically and others using online platforms; such as Microsoft Teams and Zoom. There was no need for any translation services as all the participants were comfortable with conducting the interviews in English. A maximum of 30 min was scheduled per interview, with at least 15 min set aside for online connection delays. This proved to be a vital intervention because the most common challenge during the interviews was poor network connectivity; consequently, some interviews had to be rescheduled or alternatively conducted telephonically.

### Data analysis

The ATLAS.ti 8 research software was used to analyse data through content analysis. Researchers have supported the use of qualitative content analysis to identify and categorise data into themes (Bengtsson 2016). ATLAS.ti is a computer-assisted qualitative data analysis software, which allows researchers to effectively code data and create interacting themes to interpret data (Adelowotan 2021). In addition, as supported by Paulus and Lester (2016), the use of ATLAS.ti is extensive, robust and much faster compared the manual coding process. This proved to be a viable option considering the large volume of interview transcripts from the 50 participants. The interview transcripts were colour coded into specific themes. The study guided based on previous research by Moosa-Tayob and Risenga (2022) and Jackson et al. (2016), developed a six-stage which involved familiarisation, coding, searching for themes, reviewing themes, defining and naming themes and writing the research report. As for coding, an inductive approach was followed, which allows for a bottom-up perspective, whereby the researcher uses the participants responses to generate and interconnect themes (Creswell & Plano Clark 2017; Tenny et al. 2021).

### Ethical considerations

Steffen (2016) presents that it is with uttermost importance that in any research ethical procedures and considerations must be followed. The study was registered and approved by the Research Ethics Committee at the University of Pretoria (Ethics approval number: HUM035/0820). In addition, participation was voluntary, and permission to conduct key informant interviews was obtained from all the participants, through informed consent. Thus, all the information was treated as confidential. La Rossa and Bennett (2018) add that the participant should have the legal capacity to give consent. All participants signed informed consent forms prior to the interviews.

## Research findings

The right to basic education cannot be implemented in isolation while ignoring other fundamental rights prescribed in the Constitution, education policies and ratified treaties. To fully comprehend the collected data, the researcher focussed on the six main basic education challenges on children with disabilities, based on the participants’ responses.

### Limited number of special needs schools

The major challenge raised by the participants during the research was the issue of insufficient available schools to cater for the needs of children with disabilities. These reflections were illustrated in the following interview excerpt:

‘We don’t have enough schools, very minimal, especially for children with disabilities. Since we are here in Port Alfred, you will find that the nearest centre is far away.’ (P3, NGO representative, Port Alfred)

The above-stated contribution reflects on the situation in Port Alfred, whereby there are few special needs schools available, and in most cases, these children have to travel further to the nearest school. The situation as expressed by another participant is worse in rural areas, where the only available special needs school is located in the urban city of Gqeberha:

‘For special schooling here, we only have 2 Government special schools in Gqeberha. Therefore, you can understand there’s a massive waiting list or sometimes, you know a child is on a waiting list. And obviously, there are specific criteria which you need to perform to be placed in a special school. Most children or you know, are on waiting lists in stages. Not enough special schools to accommodate all our learner’s needs. The waiting list of up to 550 learners per annum. Over 2500 children are still at home because they are intellectually challenged.’ (P5, special needs teacher, Ndevana)

As stated in the quote, the participants from a rural area expressed frustration because of the excessively long waiting list of 550 learners per annum, with over 2500 children with special needs reported to have no access to education. This similar trend was noticed in other rural areas, such as Matatiele and Nqanqarhu.

### Scholar transport

Concerning scholar transport, children with disabilities have to travel long distances to the nearest school. As a result, the child might not be able to walk every day to school, and there is an additional cost required for safe transport. These sentiments were shared by one of the participants:

‘So, to go to school, they have to travel 320 km from the nearest city. Or in some areas, the nearest school would be 60 km daily.’ (P2, community leader, Kwazakhele)

Thus, children with special needs are severely disadvantaged when they have to travel long distances to school and yet children without disabilities will easily walk or have access to cheaper public transport options when travelling to school. This view is reflected further:

‘Some of the children can easily walk. But some of the special schools are in Uitenhage, Sunshine, Moakley and Besa, so we have to go abroad. We have to drive them every day usually two loads to transport all the children. Then on rainy days the rest of the staff just have to pull in and get in a car and drive the kids.’ (P8, community leader, Nqanqarhu)

This shows that some children had to walk to special needs schools and will require assistance to make sure they reach the school safely, as some of them have to cross rivers and dilapidated bridges to get to school.

### Enrolment criteria

Apart from the limited number of special schools, the other area of contention during the research was the criteria used to enrol learners with special needs. This was expressed by one of the participants as follows:

‘But the specialised school takes children from specific ages from the age of 14, which is a school of skills basically, and that child needs to be on a Grade 5 level academically to be able to do those things. But some children do not fall on that level or fall above the percentile or below. And because of such a system they end up placed at schools that don’t have an emphasis on academics, which kind of just exacerbates their motivation.’ (P9, special needs teacher, Gqeberha)

The response shows the criteria used is the age of the child; unfortunately in this case, this would mean that children with disabilities above 14 years might struggle to get access to a special needs school. Other than age, the criteria used to distinguish disability was inconsistent. Another respondent reported:

‘And that is an enormous problem class because there are no remedial [*sic*]. There is only one special school in East London, [*name withheld*] but it’s full of kids who are significantly disabled. So, they’re either significantly intellectually disabled or physically disabled. And these kids are not they are just “slow” if you want to use that term. And the sadness for me is that the local Public School with less than a half kids will not give the kids the right standard of examination so that they get more than 50%. But in fact, they’re not achieving. They do it so that they can make themselves look better. And those kids will proceed; whereas our kids who will be performing at the correct level will not be accepted.’ (P16, special needs teacher, East London)

The frustrations expressed show various inequalities in the criteria used for the acceptance of children with special needs. The researcher was astonished by the perception in public schools, whereby the term ‘slow’ was used to describe children with special needs. Notably, during standard examinations, they were awarded free marks to proceed to the next level. The reflections show the bias used to assess children with special needs between private and public schools. In terms of assessment, the participant indicated that intellectually and physically disabled children are not assessed with the national standard of examination.

### Lack of psychosocial and expert support

The need for psychosocial and expert support helps to easily identify learning challenges and quickly prescribe for relevant assistance. This was raised by one of the participants as follows:

‘Let me give you a scenario in the Eastern Cape, we have 61 social workers and 63 psychologists, and we do not have enough people to provide psychosocial support. Even with the Learner Support agents, we have about 300 of them, so can imagine, versus the number of schools that we have.’ (P32, government representative, Bisho)

The response shows that there is lack of expert assistance concerning the large population of vulnerable children in the Eastern Cape province. Expert assistance is not limited to social workers and psychologists but involves a wide range of specialists to assist learners with special needs. This is explained by the response:

‘Many of our learners also present with comorbidities such as physical, psychological, and neurological disabilities. We have social workers, a physiotherapist, an occupational therapist, an audiologist, an educational psychologist, and a nursing sister. All these are paid for by the school because of a lack of funding from the government. There is a lack of experts in the department to offer support and guidance to children with special needs.’ (P45, NGO representative, Ndevana)

The above-stated response summarises the challenges experienced in providing expert assistance for children with special needs. It shows that there is a lack of funding to support such professionals, because many of these challenges are not only human resource related, but quality equipment is also needed to ensure that specialists operate at full capacity. The research findings reveal that these services are only available in some schools and a large percentage of rural schools do not have access to expert assistance. This assertion makes Matland’s top-down approach relevant because in this case, it will initiate the availability of funds to allow the required expert human resources to be delegated in all special schools, even in the most rural areas.

### Water, Sanitation and Infrastructure

Section 27(1)(b) of the *Constitution and Water Services Act 108 of 1997* highlights that access to clean and safe water is a basic right (Republic of South Africa 1996). Access to basic education takes into consideration the state of sanitation and hygiene, which includes the availability of toilets in schools, provision of water and hygiene. However, one of the participants expressed their frustration on the state of sanitation:

‘You would never say that we have schools that have pit latrine toilets. So, for me, it’s just a little sad that we were fixated on optics and not producing tangible results in education in the province.’ (P30, community leader, Komani)

In addition, another participant highlighted that:

‘But we’re still sitting with over 1000 schools with pit latrines. The second timeline was on school infrastructure, we still have hundreds of schools with improper infrastructure, with poor quality materials from zinc, asbestos, and all those things.’ (P28, NGO representative, Mthatha)

The Sanitation Appropriate for Education (SAFE) programme aimed at eradicating all pit latrines toilets by 2023 has yielded little progress, as the Eastern Cape province still has the highest number of schools with pit latrine toilets (Amnesty International 2020). The extent of socio-economic inequalities between urban and rural areas is magnified in these findings as areas in more urban provinces (for instance, Gauteng, Northern Cape and Western Cape) have no pit latrine toilets recorded in the schools. Human Rights Watch (2021) criticises the Government of South Africa for its failure to implement safety measures for children with disabilities.

In South Africa, rural areas mostly lack the social and economic viability needed to sustain technological improvement (Cristobal-Fransi et al. 2020; Hall 2019). Section 28(2) of the Constitution recommends that the interests of the child are paramount; therefore, the safety of a child at school is significant. However, issues pertaining to lack of infrastructure development were cited during the research:

In terms of the Norms and Standards, by 29 November 2016, all schools must have access to some form of power supply, water supply and sanitation. Schools entirely made of inappropriate materials, such as mud, metal, asbestos or wood, must be replaced with new schools. In the Eastern Cape province, a lot of our work has been around creating accountability that’s linked to the law of norms and standards. (Equal Education 2016:5)

In terms of Section 4(3)(a) of the *Norms and Standards Act*, ‘all schools built entirely from mud as well as those built entirely from materials such as asbestos, metal and wood must be prioritised’. Infrastructure is not only limited to the building materials but also includes the school structure to improve the mobility of children with disabilities. Such as in the case of wheelchairs, the participant stated that:

‘The policy says we should have an inclusive education, but on the ground, they cannot accommodate even a child with a wheelchair, let alone a child with an intellectual disability.’ (P23, NGO representative, Seymour)

The provisions suggested should also include ramps, walkways, restrooms and extra space to accommodate the free movement of wheelchairs in schools. In this way, the right to basic education includes providing appropriate school infrastructure, which is safe and does not endanger the life and welfare of children with disabilities (Du Plessis & Mestry 2019; Grimes et al. 2023).

### Coronavirus disease 2019 pandemic

The first case of COVID-19 in South Africa was recorded on the 5th March 2020 (Giandhari et al. 2021; Pillay van Wyk et al. 2020). In response to the high infection and death rate, the Government of South Africa declared a state of emergency in accordance with Section 37 of the Constitution and the *State of Emergency Act 64 of 1997*.

The COVID-19 pandemic was cited as a major challenge by 72% of the responses. This was mentioned by one of the participants as follows:

‘Because of such challenges, we are left behind, and with physical learning, because of Covid, they’re only allowed to attend classes once or twice depending on the lockdown restrictions. In some cases, students have gone months without access to learning material.’ (P6, special needs teacher, Chintsa East)

While these challenges prompted the need for innovation through online learning. Special schools, especially in the rural areas of the Eastern Cape province, were not prepared for these changes. As illustrated by one of the participant:

‘Currently, we are working in a climate that uses the hybrid model which also physical and virtual learning. The challenge is that working with learners from previously disadvantaged backgrounds means their economic status does not necessarily allow them to participate in virtual learning because of various reasons such as, they might not have cell phones or laptops and limited access to internet.’ (P38, special needs teacher, Nkwenkwezi)

Dube (2020) remarks that during the COVID-19 pandemic, many schools were limited because of the lack of access to online learning. This proved that the chasm between the ‘haves and the have-nots’ especially when considering the socio-economic inequalities in South Africa is actually true. Thus, following the recommendations of the Republic of South Africa (2023), the government calls for the need to ensure that online learning initiatives are fully accessible for learners with disabilities.

## Discussion

Overall, the research found that children with disabilities in the Eastern Cape province faced multiple barriers to education, which are largely operational at the education system level. The study summarises the challenges facing learners into four key categories, namely, capacity, mobility, resources and disaster response on children with disabilities. Because of such limitations, findings have shown that children with disabilities are less likely to access education (Mizunoya, Mitra & Yamasaki 2018; Simo Fotso et al. 2018; Sumbane et al. 2023).

The capacity-related challenges include a limited number of special needs schools and enrolment criteria. Zwane and Malale (2018) similarly revealed that the lack of education facilities for children with disabilities contributes towards overcrowding in the few available schools. While the aspect of overcrowding was not reflected in the participants’ responses, if the issues presented in this study are not urgently addressed, the quality of education for children with disabilities is compromised.

Moreover, the research showed that the limited number of special needs schools challenges is intertwined with mobility challenges, because children with disabilities had to travel long distances, going as far as 60 km to the nearest school. Previous studies have documented scholar transport challenges in the Eastern Cape province including backlogs in transport provision and the use of unroadworthy vehicles to transport children (Chirowamhangu 2023; Kota et al. 2021). This has been extensively articulated in existing literature (Duri & Luke 2022; Kett, Cole & Turner 2020; Park & Chowdhury 2018). In addition, barriers are not just limited to the availability of scholar transport but should prompt continuous advocacy to address negative attitudes by transport drivers and aspects pertaining to the safety, security and cost of scholar transport for learners with disabilities (Calle et al. 2021; Gogiashvili et al. 2019).

The resource-based challenges were echoed in a research focussing on children with autism spectrum disorder (ASD) in Limpopo, which similarly to the Eastern Cape is predominantly a rural province with extensive poverty levels (Sumbane et al. 2023). This category of barrier includes lack of water, poor sanitation, dilapidated infrastructure, psychosocial and expert support. In their assessment, Equal Education (2016) and Odeku (2022) revealed that primary schools in the Eastern Cape province are exposed to pit latrine toilets with poor hygiene and broken sanitation infrastructure, which are issues of concern for learners with special needs. Furthermore, with regard to poor physical infrastructure, Mpu and Adu (2021) on the South African perspective state that poor school physical infrastructure is one of the major hindrances faced by learners with disabilities. In their comprehensive research of the Eastern Cape province, they highlighted that most school buildings had long staircases, which could not be accessed by learners with disabilities (Mpu & Adu 2021).

Psychosocial and expert support involve assistance from comprehensively trained personnel with best inclusive practices (Amod 2015; Engelbrecht et al. 2015). This perspective is highlighted in previous literature on inclusive education (Forsberg & Schultz 2023; Pillay, Patel & Setlhare-Kajee 2023). As a researcher, I concur with this view from a structural functionalist perspective on disability, which advocates for the awareness of inclusive education on family and community structures.

In relation to disaster response on children with disabilities, specifically with regard to the COVID-19 pandemic, the World Health Organization (WHO) confirmed that learners with disabilities were impacted more significantly by COVID-19 (WHO 2022). In view of online learning, Tremmel et al. (2020) show that because of COVID-19, there was little planning and resources to accommodate remote learning, especially in rural localities. Moreover, online learning for learners with disabilities was not only limited to the mode of instruction, but scholars extend this scope to incorporate the frequent physical contact required to carry, lift and feed students while learning (Kuper et al. 2020). Consequently, when responding to disasters, such as in the case of COVID 19, governments should allocate all necessary resources and prioritise communication with all relevant stakeholders (Beckmann & Reyneke 2021; Magedi et al. 2023).

Therefore, the Matland’s top-down approach to policy implementation helps to reduce the gap between the actual inclusive policy and practice. This is achieved by clearly defining the set goals on policies to support learners with special needs. The literature in this research shows that there is no lack of policy on children with special needs, that is, from the *South African Schools Act* to the White Paper 6 on inclusive education. But there are not clear-cut guidelines and timelines to address the challenges raised in this research. For example, the White Paper 6 from its inception had a 20-year provision to reach 380 special schools (Department of Education 2001), but there are no guidelines and timeframes to address the resource-based and disaster response challenges. The COVID-19 pandemic was meant to be a wakeup call, that if no tailor-made policies are in place, the most vulnerable are affected. Donohue and Bornman (2014) support that reducing the ambiguity in policies promotes the inclusion of children with disabilities in education.

### Limitations

The use of online and telephonic methods of collecting data was a challenge, considering the poor network connectivity and lack of Internet access. As a result, some interviews had to be rescheduled. Furthermore, the study was conducted at the peak of the COVID-19 pandemic, and because of lockdown measures, some participants had an intensive workload. Moreover, because of travel restrictions, other participants were in different time zones, and the interviews had to be re-scheduled accordingly. It was difficult to accommodate all interviews; therefore, some interviews were conducted late at night or early in the morning based on the participants’ preference. To assist with time management during data collection, the interview schedule was changed depending on the participants’ responses and the thematic approach helped to navigate through the questions.

In terms of the language of communication, though all the participants were comfortable to conduct the interviews in English, there were few instances whereby the questions had to be restructured to assist the participants to best express themselves. Scholars prescribe that the quality of interview depends on the skill of the researcher to listen carefully to the participants’ responses and navigate the responses focussing on the research questions (Cairns-Lee, Lawley & Tosey 2022).

### Recommendations

In response to the participants’ responses, the following recommendations are proposed to address the basic education challenges of children with disabilities in the Eastern Cape province:

- To improve engagement with local communities and NGOs, especially in the rural areas, the voices of vulnerable people in marginalised areas deserve to be heard.
- Provincial government Department of Basic Education must put measures to delegate and account for resources to support learners with disabilities.
- Develop mechanisms to monitor and evaluate progress on the implementation of the White Paper 6 and other regional and international treaties.
- Construct special needs schools improve the quality of infrastructure, provide scholar transport and develop working opportunities to attract expert support staff to ensure improved service delivery in rural areas.

## Conclusions

The article using the case study of the Eastern Cape province reflected on the challenges facing children with disabilities with regard to access to basic education in South Africa. The findings show several challenges including the limited number of special needs schools, enrolment criteria, lack of psychosocial and expert support, scholar transport, water, sanitation and infrastructure and the COVID-19 pandemic. The research acknowledges Matland’s top-down approach as a roadmap to effectively implement policies to support children with disabilities. This helps to provide clarity on the context and accountability measures on implementing the White Paper 6 on special needs education.
